# Who is a mobile man ‘at risk’? Masculinity, work-related mobility, and HIV-related risks in Uganda and South Africa

**DOI:** 10.1016/j.jmh.2026.100414

**Published:** 2026-05-01

**Authors:** Martin Mbonye, Janet Seeley, Julie Fox, Emily L Webb, Andrew Medina-Marino, Eugene Ruzagira, Thabang Manyaapelo, Seluleko E. Ngcobo, the Mobile Men Study Team

**Affiliations:** a MRC/UVRI and LSHTM Uganda Research Unit, Entebbe, Uganda; b Africa Health Research Institute, KwaZulu-Natal, South Africa; c London School of Hygiene & Tropical Medicine, London, UK; d King’s College London, London, UK; e Desmond Tutu HIV Centre, University of Cape Town, South Africa

**Keywords:** Migrant workers, Masculinity, Pre-exposure prophylaxis, Sexual behaviour, Sub-Saharan Africa, HIV prevention

## Abstract

Men who move for work (e.g., taxi drivers, fishermen, labourers) are often described as a homogeneous group at equal risk of HIV acquisition. This framing often masks important differences between men. Using data from rapid ethnographic assessments and a baseline survey in two settings in South Africa and one in Uganda, we examine how sexual risk- taking among men who travel frequently for work is shaped by mobility. We describe the different mobility patterns of men who are engaged in fishing, driving taxis and trucks and motorcycle-taxi riders, showing how these patterns interacted with reputational and respectable masculinities to shape sexual risk. The availability of alcohol and sex workers while away from home offered opportunities for engaging in opportunistic sex, particularly among younger mobile men drawn to building their reputation as a sexually-active man. Other factors such as older age, relationship status, fatherhood, time in the place and previous risky behaviours, shaped the enactment of respectable masculinities, especially among the older men. These findings illustrate that the link between HIV risk and mobility is understood better when looking at the different interacting factors at play in different contexts. The same mobile men may enact different forms of masculinity depending on their relationships status, social obligations and past experiences with risk, resulting in different exposures to HIV risk. Intervention approaches which target men who are mobile for work need to be tailored to address the different factors which affect risk-taking at different times in mobile men’s lives.

## 1 Background

The spread of many infectious diseases has historically been linked to the movement of people from place to place (Prothero, 1977). Preventing person-to-person contact and controlling population mobility have been strategies to prevent transmission, as in the case of SARS-CoV2 before vaccination became available (Zhang et al., 2022). Other epidemics, including of sexually transmitted infections, have been accelerated by mobile populations: the link between HIV spread and particularly mobile people has been described in many different settings (Aggleton et al., 2014; Quinn, 1994; Weine and Kashuba, 2012).

However, when mobility is given as a blanket cause of HIV acquisition, it may lead to simplistic assumptions about the source of risk. An example is the work of Randy Shilts (1987) in his account of the beginning of the AIDS epidemic in the United States. He described the role of `Patient Zero’, a Canadian flight attendant, in the spread of HIV; a role subsequently disproved because the strain of virus this man had was already in the United States before he arrived (McKay, 2017). However, the idea that a man who was mobile for work was the carrier of infection fitted with the narrative of an individual away from home engaging in risky sexual practices. That same narrative has been applied to other mobile men, including fishermen in Uganda (Westaway et al., 2007) and truck drivers in East and southern Africa (Delany-Moretlwe et al., 2014; Gysels et al., 2001; Mutie et al., 2024). Yet, as Deane et al. (2010) describe, sweeping assumptions about all members of a mobile population being equally at risk is often a limited, and sometimes prejudicial, approach to the definition of mobility. Differences between the men who move, what they do, where they go and the type of destination, are overlooked when mobility is assumed to be the cause of HIV-related risk.

The linkage between mobility and HIV acquisition has come under increasing scrutiny for being too simplistic (Aggleton et al., 2014; Camlin and Charlebois, 2019). Despite high HIV prevalence and documented behaviours that may increase one’s risk of HIV acquisition, there are members of mobile populations who recognise the risks and make deliberate efforts to protect themselves (Deane et al., 2010; Kwena et al., 2013). Even among those who may sometimes take risks, there can be times when they are more cautious (Sileo et al., 2018). Types of mobility are varied and cannot be reduced to a single indicator such as “number of nights away from home”. Rather, other factors including where someone stays, who they stay with, and/or how often they visit, may influence their risk more than the fact that they are mobile for work (Colebunders and Kenyon, 2015). As Deane et al. (2010, p. 1461) observe, we need a thorough understanding of who moves, the specific form of mobility engaged in, why they move and the characteristics of the sending and receiving areas.

The need to find work and sustain an income is an integral part of men’s sense of manhood and helps them earn respect in their family and community (Siu et al., 2013; Wyrod, 2008). Wilson (1969) proposed two types of masculinity: reputational and respectable. Reputational masculinity is associated with behaviours such as sexual experimentation, alcohol consumption and frequent expenditure on leisure. Respectable masculinities are associated with societally acceptable behaviours, including working hard to provide for one’s family, getting married and being faithful to a sexual partner. Subsequent work using these categories has shown that men may express different forms of masculinity in different contexts (Mbonye et al., 2022; Siu et al., 2013), which may help us to understand why men who are mobile for work may engage in sexual risk-taking in some circumstances and not in others (Nakazibwe et al., 2022; Wyrod, 2008). In this paper we use the concepts of respectable and reputational masculinities to reflect on the different factors that promote differences in sexual risk behaviour for men who are mobile for work.

We describe the mobility patterns and masculinities enacted among by men who travel frequently for work, drawing on data from Uganda and South Africa. We describe the heterogeneous experience of work-related mobility and HIV risk behaviours among seemingly similar men and draw on different aspects of reputational and respectable masculinities to examine risk and HIV acquisition.

## 2 Methods

Between November 2023 and July 2024, we conducted rapid ethnographic assessments (REA) prior to a planned multi-setting, open-label randomized implementation trial that provided men who are mobile for work with informed choices for HIV pre-exposure prophylaxis (PrEP). For the purposes of the trial, a man was classified as ‘mobile for work’ if he had travelled for work or to find work in the past six months and had spent at least one night away from home for work-related purposes. Eligibility criteria for the trial included: 1) cisgender men, 2) aged ≥18 years, 3) HIV negative.

### 2.1 Study settings

Study locations were selected based on a history of high HIV burden and in consultation with local stakeholders. In Uganda, Masaka District was selected, while in South Africa, Buffalo City Metro (BCM) and uMkhanyakude Districts were selected. Masaka District is located on the northwestern shores of Lake Victoria. The District has an estimated population of 115,000 (UBOS, 2024), with an economy driven by agriculture and fishing. The estimated HIV prevalence in fishing communities around Lake Victoria ranges from 21 percent to 40 percent (Nakamanya et al., 2023). REA were conducted in four communities. One community is predominantly a fishing village with bars, restaurants and shops servicing those engaged in fishing activities. The second is a fishing village located approximately six kilometres from the first fishing community. It includes a mainland ferry port serving the islands of Lake Victoria, where fishermen and travellers often stay overnight. The third site is a transit town with travellers coming from western Uganda. There are several guest houses and bars/restaurants which attract many sex workers. The fourth site is an island located approximately four hours from the mainland and close to the border with Tanzania. It is a popular location for fishermen during the fishing season and serves as a transit route to other islands.

The BCM, located in Eastern Cape Province, has an estimated population of 975,000 people; in 2023, the estimated HIV prevalence and incidence among men aged 25–49 years living in Eastern Cape was 16.2 percent and 0.58 percent, respectively (Johnson and Dorrington, 2023; Johnson et al., 2017). REA were conducted at three sites. The first site is a taxi hub located within the East London city central business district. The second site is a large taxi rank located approximately 20 km from the first site. Taxis departing from both taxi sites travel widely within BCM and regionally. The third site is a construction site located approximately 3 km from the taxi rank. Both are located within Mdantsane Township, one of the largest townships in South Africa, with a population living mainly in low-cost and informal housing. uMkhanyakude District, located in KwaZulu-Natal Province, has an estimated population of 738,000 people; in 2023, the estimated HIV prevalence and incidence among men aged 25–49 years living in KwaZulu-Natal was 20.5 percent and 0.38 percent, respectively (Gareta et al., 2021; Johnson and Dorrington, 2023). REA were conducted at two sites. The first is a taxi hub in a town of approximately 30,000 people. This taxi hub serves both local and long-distance travellers, some of whom are from other parts of South Africa. The second site is a small town of approximately 3000 people. The town has a timber yard on the edge of the town and a service station/truck stop about 15 km from the town used by long-distance travellers, including truck drivers.

### 2.2 Data collection

The rapid ethnographic assessments documented the daily living and work arrangements of mobile men in the study areas. We assessed the existing HIV prevention and care landscapes in the communities where prospective participants stayed and gained some understanding of the types of mobility the different men experienced.

The REA approach involves a mix of qualitative methods that are organized in a sequence (Manyaapelo et al., 2025). The approach involves researchers being immersed in a setting for a period of 12 to 15 days to gather information about the whole community, focusing broadly on the topic of the research to be pursued in a subsequent trial or study. REA data from each site therefore provided information on the context through which the study team could understand mobility and HIV risk from the perspectives of mobile men and other community members.

Data were collected using observations, spiral/transect walks, individual conversations, in-depth interviews, natural group discussions and focus group discussions (Bond et al., 2016; Manyaapelo et al., 2025; Mugisha et al., 2019). Prior to field work, community engagement activities were conducted with key stakeholders to discuss the study and how it would be approached. During these meetings, the research team was introduced to community leaders. These engagements further sought to help plan community entry and gain permission to conduct the study.

A topic guide, focusing on mobility and perceptions of sexual risk, as well as prompting a general discussion about day-to-day life in the community, was used for both the in-depth interviews and group discussions. For observation activities, we used a flexible checklist, based on Bond et al. (2023), which was updated as more information on the site emerged during field work. Team debriefings were held after each field work session to discuss emerging themes and to plan the next day’s activities. Informal conversations with community members, conducted spontaneously whenever the opportunity presented, were important additional sources of data.

Written informed consent was obtained for those who participated in formal interviews and group discussions. Verbal consent was obtained for informal conversations. Local leaders approved of and provided verbal consent for observational work undertaken in public places (i.e., markets, road junctions, transport hubs).

The findings presented in this paper are drawn from 27 group discussions, 58 in-depth interviews, ∼300 informal conversations with men and women, and observational work in the study communities. The sampling was purposive to capture the variety of characteristics of the men and their work in a particular setting. For focus group discussions and in-depth interviews, we collected data from information-rich cases in each setting until we reached a point where we were not collecting new information. This point was arrived at after regular team meetings reviewing the data until thematic saturation had been reached.

To provide further context for the qualitative data and analyses, we also include descriptive information on mobility from baseline surveys conducted with study participants enrolled as part of the implementation trial in the same settings. Survey participants were asked about whether they had stayed overnight in an area other than their usual area of residence in the last six months, and for how many nights if so. They were also asked whether they had stayed overnight elsewhere in the last three months, and in how many different places if so.

### 2.3 Data analysis

Analysis of the qualitative data was iterative and was conducted concurrently with data collection. There were two stages: (1) a rapid analysis from which we produced an overview report on each site. This report was used in preparation for setting up the trial, informing the location of mobile clinics and several factors to consider in the operation of the recruitment of participants. (2) A finer thematic analysis when all interview and discussion group transcripts, field notes, diagrams and maps were included.

A shared coding framework was developed collaboratively by the teams across the sites based on the research questions and themes that emerged from the rapid analysis. The framework consisted of codes that focused on the different patterns of mobility, the different jobs men engaged in, masculinities in different contexts, and perceived risks and protection mechanisms. This framework was used to organise data into categories focused on mobility patterns as well as themes that emerged from the study findings.

Differences and similarities in the data across the Ugandan and South African sites were compared as coding progressed and then organised to construct a picture of how mobility was described across the study communities, with attention paid to the local context. Any uncertainties during the coding process were addressed through team meetings to arrive at a consensus. This iterative process allowed for the refining of the coding framework without losing the site-specific context during the analysis.

For the baseline survey data, we conducted descriptive analyses to summarise participant characteristics overall and by setting. Continuous variables with skewed distributions were summarised as median and interquartile range, and categorical variables were summarised as frequency and percentage. These measures were compared between groups using the Kruskal-Wallis test (continuous variables) and chi-squared tests (categorical variables).

## 3 Results

During the baseline survey for the trial the men were asked if they had stayed overnight in an area other than the one they were in for work in the last 6 months. All 400 participants answered yes because this was a requirement for enrolment into the trial, and the question in the survey was intended to be confirmatory. However, when asked how many nights during that period they had stayed outside the community where they were staying now, the median among men in South Africa was two nights (interquartile range 2-5 nights), while in the Ugandan fishing site the median was 30 nights (interquartile range 14-60 nights) (p-value for difference between settings = 0.001). Among the men who said that they had moved residence in the last three months, most (81 %) at the East London sites said they had stayed in one to two different places, for KwaZulu-Natal there was a more even spread with about 25 percent each saying two, three, or four to nine different places, with 14 percent saying one. In Uganda most men (78 %) had stayed in three or more places. These data give an indication of differences across sites (p-value <0.001). The qualitative findings indicate that mobility patterns varied among the men and risk depended on work type, the stage of life, the relationship status and the social context within which men moved*.*

We present the qualitative findings in two sections. The first describes mobility patterns across the sites. In the second section we describe the interplay between masculinities of reputation and respectability showing how each may shape the risk of HIV acquisition among the mobile men.

### 3.1 Mobility patterns among Ugandan and South African men

There were different forms of mobility across the study sites depending on the duration, purpose, frequency, and distance from home. These differences shaped the degree of conformity to respectable or reputational masculine norms. All the men saw mobility as a route to achieve the means to earn money and thus allow them to play a masculine role of provider. Across all sites, some men reported being predominantly daily commuters, travelling for work from a particular destination they called home to another and returning the same day. Such men included short-distance truck drivers in South Africa, traders who stayed in one place but conducted business in another, some categories of commercial motorcyclists (called boda-bodas in Uganda) and taxi drivers who drove specific routes and often returned home the same day. Others included some categories of salaried employees including health workers, policemen and soldiers who often returned to designated homes after a day’s work. However, sometimes these men would stay away from home because work was not always predictable:

*Most of the time, I drive around East London. It’s rare that I go outside the city unless the taxi owner asks me to take a group somewhere out of town. That only happens occasionally. Most of the time, I drive locally.* (IDI-East London-South Africa, taxi driver, male, 54 years old)

Others undertook a mix of local and more distant work, depending on the circumstances. This is illustrated by a South African taxi driver, who usually travelled only short distances:

*I used to be tired a lot, I used to drive far, now I just do local, and mostly I don’t even drive, I just come to see if the young boys are doing the job right and sit there the whole day. When I go home, I do a few kilometres of running, and that makes me want to have sex all the time. I think it’s the running and not being on the wheel like I used to.* (Conversation-East London-South Africa, taxi rank manager, male, 54 years old)

Some men travelled away from home and would stay away for a few days to a week. In South Africa this included long-distance truck drivers and some categories of taxi drivers. The younger men appeared more flexible and open to unpredictability if it created more earning opportunities:

*I drive local and long-distance, for instance I am from Cape Town today and I am going back again, around 17:00 I should be leaving here. It depends how many days you stay, because you might arrive there and get lucky and get a load coming this side from someone else who was not feeling like driving all the way to this side then you would take that load, you can stay for about a week, otherwise you can stay for as long as a week, three days or even two days you see*. (IDI-East London-South Africa, taxi driver, male, 36 years old)

Those taxi drivers who travel to destinations over 600 km, often slept at the destination. The taxi loading rotation system is organized in such a way that it may take up to three days to get passengers to make up a full load before driving back to their home station. The work pattern was not always predictable:

*Eh, it depends on how quickly loading is on the stands. We sometimes offload and quickly go back to where we came from with another load, but we normally don’t stay for more than 4 days.* (IDI-KwaZulu-Natal-South Africa, taxi driver male, 31 years old)

There were some jobs which followed a seasonal pattern, for example the fishermen on Lake Victoria in Uganda. These fishermen specialized in different types of fish which were available during certain seasons, and this dictated how they structured their mobility. Some fishermen specialized in the small silver fish locally known as *mukene* in the Luganda language, while others went for the middle-sized fish called tilapia or *ngege*. Others fished for Nile Perch (*mpuuta)*. When the fish harvests were plentiful there was an increase in the number of (mostly) male workers at the landing site. Some of the richer traders owned trucks with freezers and these could be seen parking in a specific area of the landing site, carefully packing the fish onto their trucks during the season. These vehicles increased in number when the demand for a particular fish type increased and reduced when the desired fish was not available. As well as the truck owners, this work gave opportunities to seasonal workers packing the fish onto waiting vehicles of varying sizes and bicycles to transport to in-land markets. The drivers of the vehicles that transported the fish from the landing site, and the traders who they came with, often had to spend nights at the site while waiting for the fish to be sorted. In some cases, some men who came in to participate in the fishing value chain spent months in an area if the harvest lasted for a long time. This category included traders in fish, those who bought fish to feed their domestic animals, those who pushed and pulled the boats in and out of the water, and those who repaired nets and boats. As in South Africa, work patterns in Uganda determined how long a man stayed in a particular place before returning home:

*The fishermen are highly mobile, and this includes those who come to buy silver fish in large quantity (from Masaka). These spend time in the landing site before they go as they wait for their desired quantities. They usually spend a week [but sometimes] months in different places.* (IDI-Uganda, lay health worker, female, 49 years old)

Sometimes fishermen in the Ugandan site travelled to remote islands, other times they moved to other landing sites a distance away in search of fish. In such cases, they used boats of varying sizes and quality but it was not common for these fishermen to have life jackets. For some men, the ability to navigate the lake water without the life jackets was a sort of validation of their toughness as men, which enhanced their masculine reputation among peers. To gain more courage, they took alcohol when they went to fish and continued whenever they returned to the shores as a way of celebrating their safe return.

For some men, if they were able to establish good relationships with boat owners or traders, they may be regular visitors to the same site for part of the year, leading them to establish a new home there.

Some of the people at the fishing sites reported that they were from other countries neighbouring Lake Victoria such as Tanzania and Kenya. Others were from Rwanda and the Democratic Republic of Congo who had permanently settled in the community and integrated well with the other people. In addition, there were refugees who had been displaced by conflict and fled to Uganda from neighbouring countries like Burundi and Rwanda. These were brought into the fishing community from camps to provide cheap labour when demand was high. They did not speak the local language, which affected their integration into the community and so they kept to themselves. A refugee contact person explained:

*They come and when the season is done, they go back. Like now [January 2024], when the season for silver fish is done, they go, but in February they come back. Because for them they are guided by the moon and sun. For example, the Barundi (Burundians) go back to their camps (refugee). And when the season starts, the contact person calls them to come back. And when the season is done, they go back to their camps.* (IDI-Uganda, bar manager and refugee contact person, male, 25 years old)

Although most mobility was work-related, during work the men established relationships with families elsewhere who they would visit from time to time. All the men in both countries were affected by the mobility linked to events such as a burials, entertainment, sports, religious or political activity, as well as visits to see relatives and friends. Some men had partners and children in rural areas who they supported and who they often visited for a night or two to attend to family matters.

Another form of mobility linked to events was as an entertainer, disc jockey or host of events which some men engaged in on a part time basis, for others it was their main work. These entertainers were flexible about where they would spend the night as illustrated by one man who reflected on his work to justify why he fitted the definition of a mobile man:

*I am a mobile man, why? Because I can organize shows for a month, and go to different places every day, for example today I can be in Sembabule, the other day Ntusi, Bukomansibi [these towns are far apart] and that makes me a mobile man.* (FGD-Uganda, mixed group)

None of the men in any of the sites in both countries fitted into a single type of mobility all the time. Driven by the provider role, they looked for jobs that could earn them an income to support their families. Some undertook a mix of jobs (i.e., construction work or fishing for a period, followed by work in a market loading trucks), others worked away for a period to earn money to support them to set up a business closer to home, and some modified their work patterns because of family demands.

*We do multiple activities, this one is a master of ceremonies and at the same time a teacher, the other one is an actor and also a teacher, this one is a fisherman and also does karaoke, that’s how it is.* (FGD-Uganda, entertainers, mixed group)

In sum, the fluidity of mobility and a desire to perform important masculine roles (i.e. being a provider) encouraged men to move in search of money. Below we examine the link between men’s movements, the masculinities they enact and their varied experience with HIV risk.

### 3.2 Mobility and HIV risk: interplay between reputational and respectable masculinities

The different mobility patterns described above presented different forms of risk for different men depending on their personal circumstances and experience. The extent of exposure to the risk of HIV acquisition emerged through the ways men enacted reputational and respectable masculinities. Being away from home presented opportunities for some men to engage in risky behaviours while for others (often older men), the knowledge of the risks that sexual activities posed to their health moderated their behaviour.

The ways in which men viewed sexual risk while away from home, and their attitudes to protecting themselves from HIV and other sexually transmitted infections, were shaped by their different circumstances. Some of those men who had families, for example, spoke about the burden of the children they already had and not wanting more children as the main reason for their use of condoms with other partners. An older South African truck driver with three wives discussed how his biggest concern about acquiring HIV or STIs was from his youngest wife:

*As a married man with three wives, there is one who is the youngest of them all who concerns me the most. I can behave 100% well but I know that she is still young and impulsive, and her blood is still boiling. I worry that in my absence, she might seek excitement outside our marriage [from other partners], which could bring harm to me.* (IDI-KZN, truck driver, male, 62 years old)

Older fishermen in Uganda reflected on similar risks emanating from sexual partners at home engaging in unprotected sex during their absence. This was informed by the perception that in fishing communities, transactional sex was so pervasive that it was assumed that all women could be approached for sex:

*The calculation for the return time when a fisherman has gone for fishing is predictable. And in this area a woman isn’t for one person. Once the partner is away another takes over. I may be of good character by not engaging into such risky sexual behaviour and so if I get the HIV infection, then the cause can be from my sexual partner.* (FGD-Uganda, boda-boda riders, male)

Some men argued that if they could negotiate for transactional sex with women known to be in relationships, there was no telling whether their own partners who stayed at home could not be enticed as well.

Other older men explained that their motivation for condom-use and regular HIV testing was tied to a desire to remain healthy and avoid jeopardising their position in their family. These practices allowed them to participate selectively in sexual relationships while practicing respectable masculinity grounded in responsibility, fidelity, and control over reproductive outcomes. Remaining healthy was important to them in order to play their provider role. This was an illustration of how men could perform and maintain their social status, their reputation, as a participant in the activities of peers such as indulging in sexual activity when they were away from home. By insisting on condoms, they were tapping into a form of respectable masculinity that comes from not having children outside the relationship while also remaining HIV free. This was illustrated by a 59-year-old fisherman in Uganda:

*I test to know my status to be able to continue protecting myself. […] I make sure I always have protected sex with anyone that is not my wife. That is why I do not have any children out of my marriage. I also think it has helped me to keep negative.* (IDI-Uganda, fisherman, 59 years old)

Many of the mobile men in the sites in South Africa expressed similar concerns about protecting their health:

*What gave me interest is that I want to know my health status. I want to know because I hear that it is very important to test for HIV more than once. As I have heard on the radio, and for instance those guys who came here with the bus [a mobile health service] said to me I should test regularly although we tested you and found nothing surprising I should test again.* (IDI-East London-South Africa, taxi driver, male, 59 years old)

These older men exercised caution in sexual encounters through HIV testing and the use of condoms while still retaining a reputation among their peers as sexually active. Community members in Uganda, including mobile men themselves, observed that a high demand for HIV testing among motorbike taxi drivers in Uganda was evidence of widespread sexual promiscuity in the community. One young male motorbike rider during an informal conversation commented that: “*The reason why people persistently test for HIV is because they are unfaithful to their partners”.*

Most of the younger mobile men especially those who were yet to establish families and stable partners in one place, reported engaging in sexual experimentation, a core aspect of reputational masculinities. They seemed to enjoy the thrill of making money and spending it on women to satisfy their sexual thirst after a hard day’s work. These men in both Uganda and South Africa did not shy away from acknowledging that they rarely used condoms. One South African taxi driver spoke about the conversations they have with other peers that reflected their risk-taking behaviours:

*What I usually notice is that as young taxi drivers we encourage each other on having sex with one and the same girl. If one said they have been with that girl, then all will then want to prove a point by getting her and sleeping with her without a condom. We do not advise each other properly but drive one another towards a cliff, that is what leads us all to trouble and risk of getting HIV.* (IDI-KwaZulu-Natal-South Africa, taxi driver, male, 33 years old)

Narratives about condomless sex and the desire to enjoy this sex were common among the Ugandan fishermen as well. They often used the analogy of eating a sweet in its wrapper as a way of justifying condomless sex arguing that such a way of eating a sweet would reduce the sweetness. A man in one of the South African sites used the same illustration:

*People do not prioritize the use of a condom, most of them usually say that “have you ever eaten sweets from the plastic?” They would often ask you that, so you would tend to think of that and laugh, it is tasteless.* (IDI-East London-South Africa, looking for work, male, 59 years old)

With such perceptions, some participants and wider community members felt that the acquisition of HIV would occur when mobile men who never used condoms engaged in risky sex wherever they went. In the case of Uganda, the fishermen who were identified as highly mobile tended to attract the most scrutiny:

*But we have two categories of people who can spread the virus. Those are fishermen because they move from here, go to other landing sites to fish and they come back. They can spread it or bring it. We have those who come to purchase fish, one might come with it and spreads it. Or he or she gets it from here and takes it somewhere else.* (IDI-Uganda, landing site manager, male, 49 years old)

However, this stereotype of the two groups masks the fact that while many young men spoke proudly of having frequent condomless sex, they also expressed worry about the risk they were putting themselves at. During data collection across the sites, we were often asked for HIV testing service advice and during informal conversations, those asking would say that they wanted to see if their recent sexual history had exposed them to HIV.

Taxi drivers in South Africa sites spoke about how they always consume alcohol after work as part of socializing with other drivers and peers. The consumption of alcohol and available willing females in alcohol selling places increased their chances of sex, often without a condom.

*Yes, alcohol does have an impact because someone’s mind after drinking doesn’t work normally, and you want to do things now even though other things require more time. Sometimes it happens that you meet a lady and after drinking you want to have sex with her now, but you don’t know much about her. If you quickly do the thing it is not easy to use a condom.* (IDI-KwaZulu-Natal-South Africa, taxi driver, male, 32 years old)

Alcohol consumption was clearly a factor in risk and yet it was a major ingredient in the entertainment that men enjoyed. The alcohol selling points were also target spots for female sex workers who were sure of getting clients willing to pay for sex after consuming alcohol. This was the case in both the Ugandan and South African sites. This is illustrated by a taxi driver from South Africa:

*There’s a lot of carelessness among people in our surroundings, even in taxis as we drive around. Especially people going to the Transkei side, there are spots they call “tsimbi zemfene”, where there are prostitutes. People go there with money to buy sex. That’s one of the risky behaviours. Even in our own community, people drink alcohol and end up not using condoms. That’s what puts them at risk of getting infected with HIV.* (IDI-East London-South Africa, taxi driver, 54 years old)

In Uganda female sex workers were, like some of the fishermen, seasonal workers following the men to different fishing sites wherever they travelled. In South Africa, the resident sex workers came to visit the taxi drivers in the evenings after they had parked:

*They visit us in our taxis maybe at night when sleeping, they come and knock asking if we want sex or not. Sometimes you find that I desire sex and ask them to get inside the taxi and if I find her attractive, we then have sex and let her go afterwards to continue with her business.* (IDI-KwaZulu-Natal-South Africa, taxi driver, male, 41 years old)

Some of the advertising for sex was on social media where the women and potential clients could engage and organise physical meetings:

*There are groups on Facebook called “Bheja, utye xxx” (bribe, eat, in xxx). They sell through those groups. They post things like, “Is there anyone who wants to mingle?”[mentions a specific unit in BCM]. Then a guy will comment, saying he's available. That’s how they end up meeting.* (IDI-East London-South Africa, looking for work, 23 years old)

Besides the risk from infections, men in all categories of work described the risk of accidents on the road or at construction sites, or drowning if working on Lake Victoria. The fear of drowning was ever present among fishermen and those who regularly used water transport. The risks in the wider environment in which men lived and work, not only during work but from violence or theft in the places they stayed, provided the context for the ways in which the risk of HIV acquisition was viewed.

Overall*,* the findings illustrate that mobility alone does not fully explain HIV acquisition risk among men who move for work. Risk is the product of an interaction of particular patterns of mobility and norms of masculinity, age and position in life, and the local context.

## 4 Discussion

Our findings show that the enactment of reputational and respectable masculinities in specific contexts of mobility shapes the understanding of HIV risk among men who are mobile for work. We demonstrate that despite some differences in the type of work done and mobility routes in Uganda and South Africa, the interaction between mobility, masculinities and stage in life often presented similar differences in HIV risk patterns. Mobility for work is common in both Uganda and South Africa, particularly in settings where salaried employment is limited and men must travel in search of work (Sikweyiya et al., 2022; Wyrod, 2008). Mobility is an important avenue to expand earning opportunities which allows men to perform their masculine roles. As they move, many men engage in activities embraced by male peers in the destination places (i.e., masculinities of reputation) (Mbonye et al., 2022; Siu et al., 2013).

However, they are also conscious of the consequences of the behavioural risks that enhance masculine reputations, which explains the precautions they may take (Gibbs et al., 2015; Siu et al., 2013). We build on existing literature by unpacking mobility and showing how different forms of mobility present different opportunities for performing respectable and reputational masculinities. This helps us understand better why HIV risk can be different among similar populations.

Money is a motivation for mobility but it also facilitates and sustains a mobile lifestyle that can expose men to risk (Aggleton et al., 2014). Indeed, our findings show that when most men earned money, the pressure to spend on leisure is balanced with the need to save and send money to their families. This leisure expenditure aligns with what is typical for reputational masculinities which are enacted through spending on entertainment, alcohol and purchasing sex (Siu et al., 2013). When men are away from home, the tendency to draw on peers to validate their manhood increases, which may explain why some mobile men in our study reported engagement in high-risk sex as a way of fitting in (Deane et al., 2010).

We found that some men conducted frequent HIV tests as a way of checking to see whether they had acquired HIV from their recent sexual experiences, a finding corroborated in the work of others (Bernays et al., 2021; Tiwari et al., 2020; Tumwesige et al., 2023). Alcohol consumption, which many men blamed for their condomless sex, is high among similar populations in Uganda and South Africa, and presents additional risks to men’s health beyond the acquisition of HIV (Kasujja and Kalema, 2018; Mbonye et al., 2014; Rashied, 2023; Trangenstein et al., 2018).

Some men in our study spoke about protecting their health to ensure they could engage in respectable masculine behaviours such as remitting money to support their families in their place of origin. The motivation to preserve health has been reported among other Ugandan men who adhere to HIV treatment in order to regain their health and continue with the provider role (Siu et al., 2013). Using condoms consistently may not have been motivated by protection from HIV acquisition, but rather to avoid having more children. Men recognized the value of providing for their families but are also aware of the economic burden of increasing family size (Wojnicka, 2021). While both these groups of men may be less likely to acquire HIV, they remain important to build sexual health and HIV prevention interventions around to ensure they can sustain protective behaviours, particularly if occasionally they have condomless sex, perhaps after consuming alcohol (Nakazibwe et al., 2022). In comparison, for men who regularly engage in unprotected sex, targeted PrEP services is a necessary first step, with the long acting injectable PrEP offering a promising option for mobile populations.

### 4.1 Strengths and limitations

The use of diverse data collection approaches to explore mobility and risk and engaging a range of participants residing in unique communities in both South Africa and Uganda is a study strength. The multi-site design of the study strengthens the comparative value of the analysis by allowing differences and similarities to be examined across settings.

There are some limitations. First the self-reported nature of accounts of sexual practices, mobility and risk which the analysis depended on could be influenced by social desirability especially since these were perceived to enhance a man’s masculine reputation. Some men may have overstated their sexual exploits to appear more accomplished. Others may have exaggerated the magnitude of caution taken in order to fit within the ideals of respectable masculinity. Nonetheless, overall the richness and depth of the data allowed for a nuanced understanding of the various forms of mobility, masculinities and HIV risk in the study settings.

## 5 Conclusion

Our findings contribute to a deeper and more nuanced understanding of mobility and HIV risk among men who are mobile for work. We argue that while mobility remains an important factor in HIV risk, taken in isolation it falls short of helping us fully understanding vulnerability to HIV acquisition. Men who are mobile for work are not a homogeneous group and should not be treated as such. Men who are concerned about their families at home, and who are older than their peers, may adopt protective behaviours or be more inclined to take up HIV prevention methods and messages, motivated by a desire to uphold their social responsibilities. Interventions targeting these populations can be more effective if informed by an understanding of the context of mobility, allowing for tailored approaches that address the specific challenges of engaging men who are mobile for work in HIV prevention and care programmes. Understanding the contexts in which mobile men may enact protective masculinities (based on respect) and risky masculinities (based on reputation) may further enhance the effectiveness of interventions. Finally, by framing the discussion around mobility, masculinity and HIV risk, we contribute to knowledge on the gendered, context specific variations of mobility, providing a nuanced conceptualisation of HIV risk and masculinity in migration research.

## Funding

This project is supported by the Global Health EDCTP3 Joint Undertaking and its members (grant number 101103140). The Africa Health Research Institute team is supported by core funding from the 10.13039/100010269Wellcome Trust [grant number 201433/Z/16/A]. The MRC/UVRI and LSHTM Uganda Research Unit team is supported by the UK Medical Research Council (MRC) and the UK Foreign and Commonwealth Development Office (FCDO) under the MRC/FCDO Concordat agreement and also part of the EDCTP2 programme supported by the 10.13039/100025961European Union. For the purpose of open access, the author has applied a CC BY public copyright licence to any Author Accepted Manuscript version arising from this submission.

## Data availability statement

The data are not publicly available due to privacy or ethical concerns. Access to the de-identified data set that support the findings presented in this paper are available on reasonable request from the corresponding author.

## Ethical approval

The study was approved by the University of KwaZulu-Natal Biomedical Research Ethics Committee (BREC/00,005,745/2023), the London School of Hygiene & Tropical Medicine Research Ethics Committee (LSHTM Ethics Reference: 29,902), the University of Cape Town Research Ethics Committee (HREC Ref: 496/2023) the Uganda Virus Research Institute Research Ethics committee (UVRI REC GC/127/982) and the Uganda National Council for Science and Technology (UNCST Ref: HS3099ES). Written (for group discussions and individual interviews) and verbal (for informal conversations) informed consent was taken from all participants before they engaged in any study activity.

## CRediT authorship contribution statement

**Martin Mbonye:** Writing – review & editing, Writing – original draft, Project administration, Methodology, Formal analysis, Data curation. **Janet Seeley:** Writing – review & editing, Writing – original draft, Supervision, Project administration, Methodology, Data curation, Conceptualization. **Julie Fox:** Writing – review & editing, Supervision, Project administration, Funding acquisition. **Emily L Webb:** Writing – review & editing, Writing – original draft, Methodology, Formal analysis. **Andrew Medina-Marino:** Writing – review & editing, Supervision, Project administration. **Eugene Ruzagira:** Writing – review & editing, Supervision, Project administration. **Thabang Manyaapelo:** Writing – review & editing, Writing – original draft, Methodology, Investigation, Formal analysis, Data curation. **Seluleko E. Ngcobo:** Writing – original draft, Project administration, Methodology, Formal analysis, Data curation.

## Declaration of competing interest

The authors declare that they have no known competing financial interests or personal relationships that could have appeared to influence the work reported in this paper.
